# Acute headache treatment in idiopathic intracranial hypertension: treating to the phenotype?

**DOI:** 10.1186/s10194-025-02236-4

**Published:** 2025-11-26

**Authors:** Gabriel Bsteh, Nik Krajnc, Sina Zaic, Nina Müller, Wolfgang Marik, Martin Bertich, Christoph Stapf, Klaus Novak, Stefan Macher, Berthold Pemp, Christian Wöber

**Affiliations:** 1 Department of Neurology, Medical University of Vienna, Waehringer Guertel 18-20, Vienna, 1090 Austria; 2 Comprehensive Center for Clinical Neurosciences & Mental Health, Medical University of Vienna, Vienna, Austria; 3 Department of Neuroradiology, Medical University of Vienna, Vienna, Austria; 4 Department of Ophthalmology, Medical University of Vienna, Vienna, Austria; 5 Department of Neurosurgery, Medical University of Vienna, Vienna, Austria

**Keywords:** Idiopathic intracranial hypertension, IIH, Headache, Effectiveness, Response, Triptans, NSAID, Real-world study, Retrospective

## Abstract

**Background:**

Effective acute headache treatment is essential for improving the quality of life in people with idiopathic intracranial hypertension (pwIIH). While current guidance recommends “treat to the phenotype”, robust data on effectiveness of available options is lacking.

**Methods:**

This retrospective analysis used standardized symptom diaries of pwIIH from the Vienna Idiopathic Intracranial Hypertension (VIIH) database (1-JUL-2021 to 30-JUN-2023). Three classes of acute medications (nonsteroidal anti-inflammatory drugs [NSAID], acetaminophen [APAP], and triptans) were analyzed, with NSAIDs as the reference. Headache attacks were classified per ICHD-3 as migraine (MIG), tension-type (TTH), or other (OTH). We used 2-level nested logistic regression models to analyze odds ratio (OR) of treatment response for each headache type, adjusting for covariance within individual pwIIH and concurrent medication with propensity-weighting for age, sex, and headache severity.

**Results:**

We analyzed 35,640 medication-outcome pairs from 23,507 headache attacks (45.3% MIG, 21.1% TTH, 33.6% OTH) in 156 patients (89.7% female, mean age 32.9 years). NSAID were most commonly used across all headache types (MIG: 60.5%, TTH: 69.8%, OTH: 70.7%), followed by APAP (MIG: 21.5%, TTH: 21.1%, OTH: 27.7%) and triptans (MIG: 18%, TTH: 10.1%, OTH: 12.8%). Triptans were the most effective across all headache types (OR for MIG: 4.8 [CI 3.9–6.1], TTH: 2.9 [CI 1.8–4.3], OTH: 3.1 [CI 2.2–4.3]). APAP was less effective for MIG (OR 0.81 [CI 0.74–0.90]) but similar to NSAIDs in TTH and OTH.

**Conclusions:**

Triptans are associated with better response rates than NSAID/APAP in acute management of headaches in pwIIH, particularly – but not exclusively – for migraine-type attacks. These findings support preferential use of triptans, questioning the “treating to the phenotype” approach.

**Supplementary Information:**

The online version contains supplementary material available at 10.1186/s10194-025-02236-4.

## Introduction

Idiopathic intracranial hypertension (IIH) is a condition characterized by elevated intracranial pressure (ICP) in the absence of a structural or secondary cause, primarily affecting young women with obesity [1]. While the primary therapeutic goal in IIH is the preservation of vision, headache remains the most common and often most disabling symptom [2]. Despite its importance, the acute treatment of headache in IIH is not standardized, and there are no evidence-based guidelines specifically addressing headache management in this context [1, 3, 4].

Standard therapies – such as weight loss, acetazolamide, topiramate, diuretics and invasive procedures such as lumbar puncture and CSF shunting – are aimed at reducing ICP and may offer partial headache relief, but their efficacy on headache is inconsistent and often insufficient [1, 3–8]. Importantly, the phenotype of headache in IIH frequently resembles that of migraine, and many patients continue to suffer from headaches even after normalization of ICP and resolution of papilledema [3, 9]. These findings suggest that headache in IIH may require distinct symptomatic treatment beyond ICP-lowering therapy.

In clinical practice, acute headache management in IIH often mirrors treatment strategies for primary headache disorders tailored to the individual headache phenotype – often termed “treating to the phenotype” – using medications such as nonsteroidal anti-inflammatory drugs (NSAID), acetaminophen (APAP), and triptans, although these remain off-label and are not supported by controlled studies in the IIH population [4, 10]. However, robust data on effectiveness of acute headache medications in pwIIH is lacking.

This study aims to investigate patient-reported response rates to acute headache medications in IIH exploring the rationale and current practice of phenotype-oriented acute headache treatment.

## Methods

This retrospective analysis used standardized symptom diaries of pwIIH from the Vienna Idiopathic Intracranial Hypertension (VIIH) database [11]. All patients with IIH (pwIIH) treated at the specialized multidisciplinary IIH center at the Medical University of Vienna (Austria) are encouraged to keep the VIIH symptom diary with daily entries throughout all disease phases. The VIIH symptom diary allows pwIIH to monitor headache attacks with regard to frequency, severity (selecting from a scale from 0 to 10), headache characteristics (uni- or bilateral, throbbing/pulsating vs. pressing/non-pulsating), aggravation by physical activity, associated symptoms (photophobia, phonophobia, nausea) and acute medication used. Effectiveness of acute medication (multiple entries and free text input allowed) can be selected from the following three options: “Helpful,” “Somewhat helpful”, and “Unhelpful” without a predefined time frame for treatment evaluation.

Here, we included all headache attack records from patients with definite IIH according to the modified Friedman criteria aged ≥ 18 years collected between 01-JUL-2021 and 30-JUN-2023 [12]. Records without information on acute medication and/or outcome were excluded.

Headache attacks were classified separately according to the diagnostic criteria of the International Classification of Headache Disorders, 3rd edition (ICHD-3) based on symptom profiles reported in the VIIH diary [13]. Headache episodes fulfilling the ICHD-3 criteria B-D for migraine were classified as migraine (MIG). Headaches consistent with the ICHD-3 criteria B-D for tension-type headache were categorized as tension-type headache (TTH). Headache attacks that did not meet full criteria for either MIG or TTH were categorized as other (OTH).

To ensure analytical focus and interpretability, we limited our analysis to three main pharmacological classes of acute headache medications: NSAID, APAP, and triptans. Within these classes, we excluded substances accounting for less than 1% of the total medication-outcome combinations in our dataset.

### Endpoint

Treatment response was defined as the primary endpoint based on self-reports by pwIIH. For each treated headache attack, patients rated the medication as “Helpful”, “Somewhat Helpful”, or “Unhelpful”. Only ratings of “Helpful” were classified as positive outcomes, i.e. response; all others were considered negative, i.e. no response. In case of multiple entries of headache medication, response was assessed for each medication creating separate medication-outcome combinations.

### Statistics

Statistical analysis was performed using R-Statistical Software (Version 4.4.2). As multiple headache attacks are recorded per patient, these are considered correlated repeated measures in the analysis. Each patient is considered to have a random effect on their treatment outcome, and every treatment is a conditional fixed effect based on the random participant effect [14].

We applied two-level nested logistic regression models to calculate odds ratios (OR) of treatment response separately for each headache type, adjusting for covariance within individual pwIIH and concurrent medication with propensity-weighting for age, sex, and headache severity. The detailed mathematical equations and explanations of each step are outlined in eAppendix 1.

To check for potential confounding, we conducted sensitivity analyses according to education level, papilledema, chronic headache, pre-existing migraine before IIH diagnosis, medication-overuse headache (MOH), multi-medication attacks, concomitant medication with acetazolamide, topiramate and anti-calcitonin gene-related peptide (CGRP) monoclonal antibodies (mAb), and invasive treatment. Significance level was set at a two-sided p-value < 0.05.

### Standard protocol approvals, registrations, and patient consents

The study was approved by the ethics committee of the Medical Universities of Vienna (ethics approval number: 2216/2020). As this was a retrospective study, the need for written informed consent from study participants was waived by the ethics committee. This study adheres to the reporting guidelines outlined within the ‘Strengthening the Reporting of Observational Studies in Epidemiology (STROBE) Statement.

### Data availability statement

Data supporting the findings of this study are available from the corresponding author upon reasonable request by a qualified researcher and upon approval by the ethics committee and the data-clearing committee of the Medical University of Vienna.

## Results

**Fig. 1:**
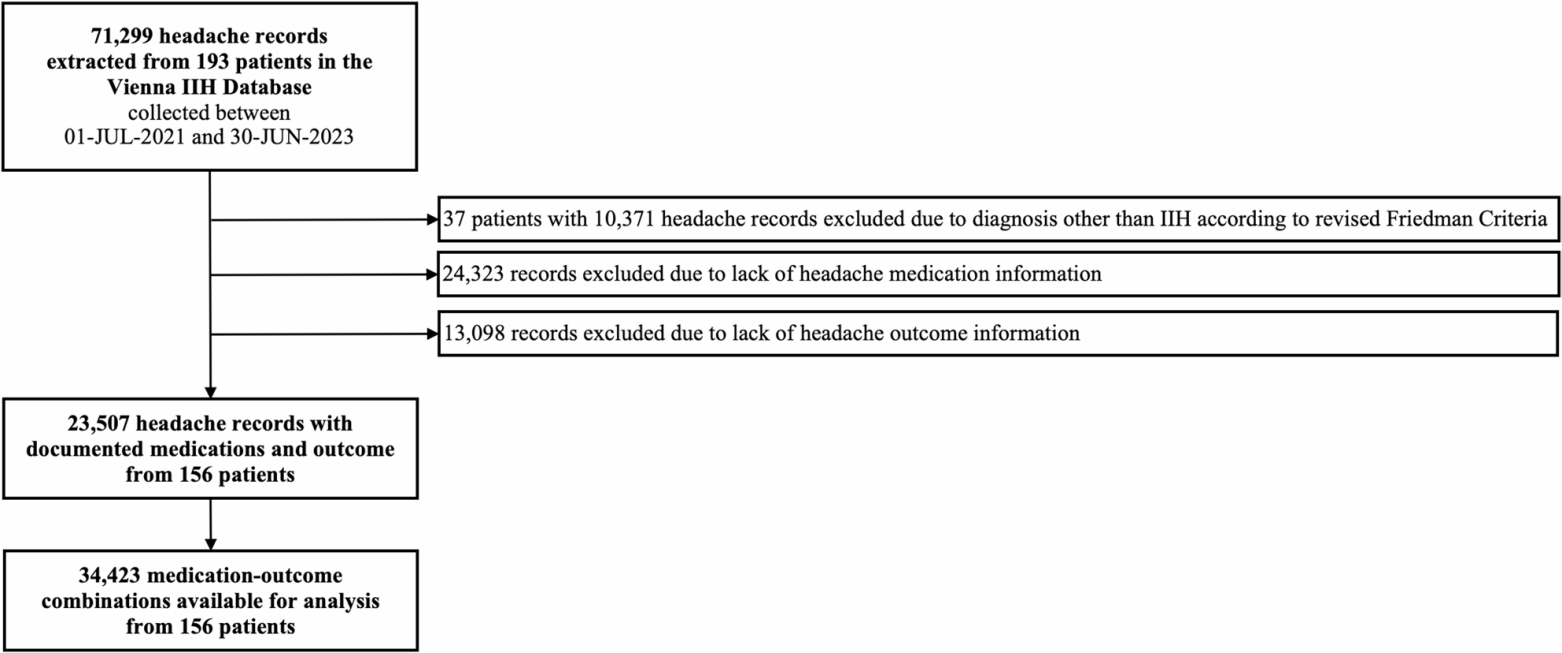
Flow chart of inclusion/exclusion process of headache records. IIH: idiopathic intracranial hypertension

We screened a total of 71,299 headache attack records in 193 individuals from the VIIH database, of which 23,507 headache attacks had sufficient data on headache medication and outcome (Fig. 1).

Of the 23,507 headache attacks included in the final analysis, a total of 34,423 acute medications were administered. Most attacks (68.2%, *n* = 16,035) were treated with a single medication, while 22.0% (*n* = 5,172), 6.3% (*n* = 1,481), 2.1% (*n* = 494), and 1.4% (*n* = 325) were treated with two, three, four, and five or more medications, respectively.

Thus, the final dataset comprised 34,423 medication-outcome combinations stemming from 156 pwIIH (89.7% female, mean age 32.9 years). Detailed characteristics of the 156 pwIIH included are provided in Table 1.

Of note, median headache frequency was 18 with a median severity of 5.5 on the numeric rating scale, 34% of pwIIH having pre-existing migraine before diagnosis of IIH, 51.9% having chronic headache and 18.6% fulfilling criteria for medication overuse-headache.

Classifying headache attacks based on symptom profiles, 45.3% were classified as MIG, 21.1% as TTH, and 33.6% as OTH. Among the 34,423 medication-outcome combinations analyzed, NSAIDs accounted for the majority of medications used in all headache categories, totalling 22,016 administrations (63.9%), followed by APAP in 7,386 cases (21.5%) and triptans in 5,021 cases (14.6%).

**Table 1:** Cohort characteristics

	*n* = 156
Female^1^	140 (89.7)
Age at diagnosis (years)^2^	33.6 (9.8)
Time from referral to diagnosis^3^ (days)	15 (1–62)
Education level^1^	
≤ 9 years of education	62 (39.7)
Highschool degree	53 (34.0)
University degree	41 (26.3)
BMI (kg/m^2^)^3^	31.8 (18.2–60.5)
CSF opening pressure at diagnosis (cm H_2_O)^3^	33 (26–59)
Papilledema grade (Frisén scale)^3^	3 (0–5)
Visual impairment^1#^	104 (66.7)
Pre-existing migraine before diagnosis^1^	51 (34.0)
Headache severity (NRS)^3^	5.5 (0–10)
Headache frequency (MHD)^3^	18 (0–30)
Chronic headache^3^	81 (51.9)
Medication Overuse Headache^1^	29 (18.6)
Time from diagnosis to treatment initiation^3^ (days)	1 (0–17)
Acetazolamide during observation period^1^	151 (96.8)
Maximum dosage (mg)^3^	750 (250–2000)
Topiramate during observation period^1^	25 (16.0)
Maximum dosage (mg)^3^	62.5 (25–200)
AntiCGRPmAbs during observation period^1^	13 (8.3)
Invasive Treatment (Ventriculoperitoneal shunt)^1^	3 (1.9)

AntiCGRP mAbs: anti calcitonin gene-related peptide monoclonal antibodies. BMI: body mass index, MHD: monthly headache days, NRS: numerical rating scale. ^1^absolute number (percentage). ^2^mean (standard deviation). ^3^median (range). ^4^calculated with chi-square test. ^5^calculated with t-test for independent groups. ^6^calculated with Mann-Whitney U-test. #defined as visual acuity ≥ 0.1 logarithm of the minimum angle of resolution (logMAR; determined by Sloan charts at distance after subjective refraction) and/or mean deviation <-2.0 in decibels (dB) in static threshold perimetry determined by 30 − 2 test with Swedish Interactive Threshold Algorithm (SITA)

**Fig. 2:**
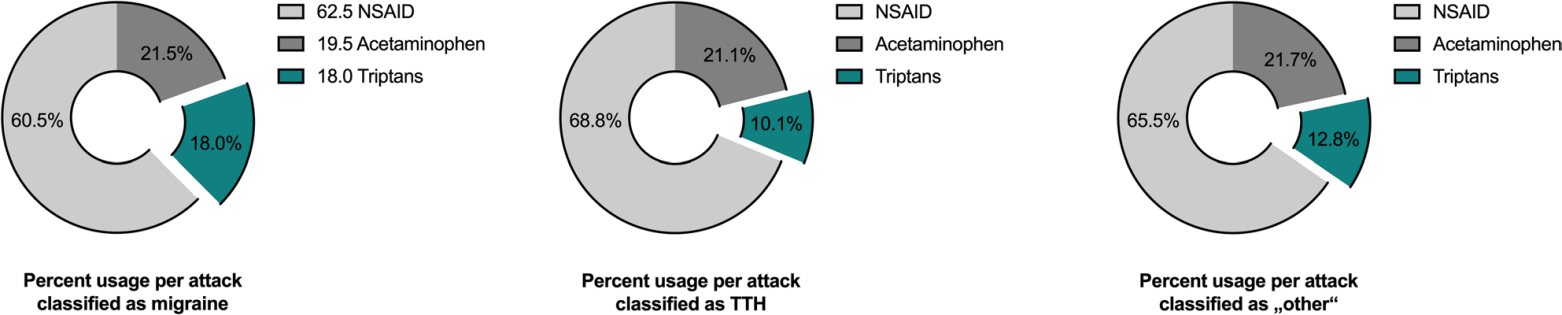
Usage of acute headache medication according to headache phenotype. NSAID: Non-steroidal anti-inflammatory drug. TTH: tension-type headache

Within the MIG group, NSAIDs were used in 9,434 attacks (60.5%), APAP in 3,353 (21.5%), and triptans in 2,807 (18.0%). Both in TTH and OTH categories, we observed a higher proportion of NSAIDs representing 68.8% and 65.5% of medication use, respectively, and a significantly lower proportion of triptan use with 10.8% and 12.8% (both *p* < 0.001 compared to MIG), respectively (Fig. 2).

**Fig. 3:**
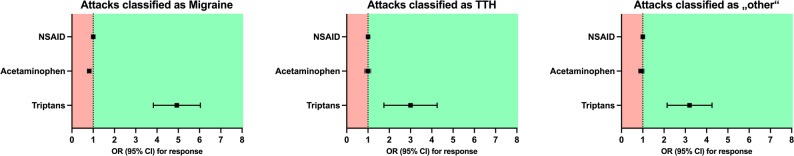
Response to classes of acute headache medication in IIH according to headache phenotype in IIH. IIH: idiopathic intracranial hypertension. OR: odds ratio. NSAID: Non-steroidal anti-inflammatory drug. TTH: tension-type headache. 95% CI: 95% confidence interval

Across the 34,423 medication-outcome combinations analyzed, 53.1% (*n* = 18,286) were rated as “Helpful”, 25.2% (*n* = 8,679) as “Somewhat Helpful”, and 21.7% (*n* = 7,458) as “Unhelpful”. Overall, the proportion of “Helpful” ratings was significantly lower in headache attacks classified as MIG (46.7%, *p* < 0.001) compared to those used in TTH (58.4%) and OTH (54.9%). Based on the multivariable regression models adjusting for within-individual covariance, concurrent medication use, age, sex, and headache severity, triptans were significantly more likely than NSAIDs to achieve treatment response in MIG attacks (OR 4.8, 95% CI 3.9–6.1, *p* < 0.0001), while APAP was associated with a lower likelihood of response (OR 0.81, 95% CI 0.74–0.90, *p* < 0.001) (Fig. 3A). Similarly, triptans showed significantly higher odds of treatment response in TTH (OR 2.9, 95% CI 1.8–4.3, *p* < 0.001) and OTH (OR 3.1, 95% CI 2.2–4.3, *p* < 0.001) (Fig. 3B-C).

In sensitivity analyses restricted to pwIIH with MOH (*n* = 29), the rate of medication-outcome combinations rated as “Helpful” amounted to 24.1% (compared to 53.1% in the overall cohort) and the proportion of multi-medication attacks was 67.3% (compared to 31.8% in the overall cohort). Correspondingly, the effect sizes for the odds of treatment response with triptans remained directionally consistent with the main analysis but were markedly smaller across headache phenotypes (OR of 2.2 [95% CI 0.9–4.1] for MIG attacks, 1.45 [95% CI 0.5–4.0] for TTH attacks, and 1.55 [95% CI 0.7–3.4] for OTH).

Sensitivity analyses regarding education level, papilledema, chronic headache, pre-existing migraine before IIH diagnosis, multi-medication attacks, concomitant medication with acetazolamide, topiramate and antiCGRP-mAbs, and invasive treatment did not significantly change the results of the regression models.

## Discussion

The present study aimed to evaluate pwIIH-reported response rates to acute headache medications exploring the rationale behind the current practice of phenotype-oriented acute headache treatment in IIH.

In this large, real-world analysis of over 34,000 medication-outcome combinations in pwIIH, we found that triptan use was consistently associated with better headache response than both NSAIDs and APAP in the acute treatment of headache attacks across all studied headache phenotypes. This effect was particularly pronounced in migraine-type attacks, where triptan use was associated with nearly a fivefold increase in the odds of achieving patient-reported treatment success compared to NSAIDs. Importantly, triptan use was also associated with better headache response in headaches with a non-migrainous phenotype, including tension-type and other headache phenotypes, challenging the conventional approach of “treating to the phenotype”. However, pwIIH fulfilling criteria for MOH seemed to have markedly attenuated overall odds for treatment response across all phenotypes with much lower differences between headache medications types.

Interestingly, the response rates observed in our study closely mirror those reported in migraine populations. A retrospective analysis of nearly 5,000,000 migraine attack records from an e-diary smartphone application found response rates of 60–78% for triptans compared to 37–52% for NSAIDs and ACAP, with a mean OR of 4.8 for response in triptans over NSAID [15]. Meta-analyses of randomized controlled trials reported 2-hour response rates of 42–76% with triptans and a 2- to 4-fold higher likelihood of response with triptans compared to NSAID [16, 17]. These effect sizes are strikingly consistent with those in our study, where triptans showed a 46.7% response rate in migraine-type attacks with a 4.8-fold increased likelihood of response.

These parallels are likely not coincidental. While IIH is primarily defined by elevated ICP, many headache features in pwIIH – such as throbbing quality, photophobia, and exacerbation with physical activity – overlap with those of primary migraine [3, 5, 9, 18]. Notably, patients often continue to experience migraine-like headaches even after ICP normalization and papilledema resolution [3, 5, 18]. This suggests that migraine-like pathophysiology within trigeminovascular pathways may be the driver behind headache in pwIIH, potentially explaining the similar response to migraine-specific treatments like triptans [5, 9, 18]. Strengthening this hypothesis, CGRP seems to contribute to headache pathophysiology in pwIIH, while there are indications that antiCGRPmAbs are effective in ameliorating headache frequency and severity [19–23].

Our results further highlight the limited effectiveness of APAP in migraine-type attacks, where it showed significantly lower odds of success compared to NSAIDs. This aligns with existing evidence from migraine populations, where APAP is generally less effective than NSAIDs or triptans in moderate-to-severe attacks [14]. Given its high usage across all headache types in our cohort, these findings may prompt reconsideration of its role as a first-line treatment option in IIH-related headache.

In line with previous studies, pwIIH with accompanying MOH seemed to experience markedly worse treatment response across all phenotypes, while differences between headache medications types appeared diminished [5, 24]. Although our study is underpowered for formal hypothesis testing in this subgroup, it seems reasonable to conclude that in pwIIH who have MOH, reduction of acute headache medication use via consequent preventive treatment is paramount and triptans should be used very cautiously given their increased risk for MOH [25].

Moving forward, the current study supports reevaluating the conventional “treating to the phenotype” approach in IIH headache management. In clinical migraine practice, NSAIDs and APAP are typically recommended as first-line treatments for mild-to-moderate headaches, while triptans are reserved for more severe attacks. This stepwise model – though cautious – may contribute to the underutilization or delayed administration of triptans [26]. Given our findings and the overlap in headache characteristics and treatment responses between migraine and IIH, it may be more effective to adopt a stratified approach that prioritizes treatment choice based on headache severity and functional disability encompassing a trial-and-error principle, rather than strict adherence to phenotype classification. However, triptan use in IIH remains off-label, and potential benefit must be weighed against safety concerns – particularly in those with MOH and cardiovascular risk factors.

Our study has several methodological strengths. The use of a prospectively maintained, structured symptom diary with daily entries allowed for phenotyping of headache attacks and reduces the risk of recall bias commonly associated with patient-reported headache data. Additionally, the application of a two-level nested logistic regression model enabled accounting for both within-subject variability and the presence of concurrent medications during each headache attack. The propensity-weighted models adjust for the influence of relevant covariables such as age, sex, and pain intensity, while thorough sensitivity analyses ruled out relevant influences of various potential confounders including papilledema, chronic headache, multiple acute headache medications, concomitant medication, and invasive treatment. This enhances the reliability and generalizability of our findings and provides a more nuanced understanding of treatment response patterns in pwIIH.

Nevertheless, several limitations are acknowledged. The design of our study was observational where treatment selection was not randomized, and medication choice may reflect physician preference or patient-specific factors. While we used propensity-weighted models to adjust for key confounders and conducted multiple pre-planned sensitivity analyses, unmeasured bias cannot be fully excluded and causality cannot be established, which would formally require a randomized controlled trial. The response ratings (“Helpful”, “Somewhat Helpful”, “Unhelpful”) were self-reported and inherently subjective, potentially influenced by patients’ prior expectations or treatment history. Although our dataset did not include standardized endpoints commonly used in clinical trials of acute migraine therapies, such as headache freedom or headache relief within two hours after medication intake, the VIIH symptom diary was designed for daily patient recordings, and this simplified scoring system likely reflects a combination of perceived effectiveness and treatment satisfaction. The symptom diaries do not capture the order or timing of medication intake within each attack, limiting our ability to adjust for time between headache onset and medication intake, and to distinguish between first-line and rescue treatments. However, most headache attacks in our dataset (68%) were treated with a single medication, and for multi-medication attacks, we adjusted for concurrent medication effects in our regression model and sensitivity analyses did not indicate a substantial impact of multi-medication attacks. Also, approximately 50% of the extracted records were excluded due to missing data on medication or outcome, which may introduce selection bias. We were unable to assess the impact of dosage and formulation on treatment response, as this information was inconsistently recorded; future studies should consider collecting and analyzing this level of detail. Another important limitation of our study is the inability to clearly distinguish primary migraine from migraine-like headache secondary to IIH. Given their overlapping clinical and pathophysiological features, the observed triptan response may partly reflect efficacy in primary migraine rather than a specific effect on IIH-related headache. This overlap introduces potential bias but also supports shared mechanisms underlying headache in IIH. Of note, sensitivity analyses for pre-existing migraine before IIH diagnosis did not substantially change the results. In this context, it is important to point out that each headache attack was classified separately rather than assigning a single headache phenotype per patient based on ICHD-3 criteria. Consequently, there is some overlap between headache phenotypes in individual patients and, thus, some diagnostic uncertainty regarding the “true phenotype”. However, this attack-level classification aligns with a “treating to the phenotype” approach, allowing for a more nuanced assessment of medication response across attack types and offering practical insights into how patients with IIH might best tailor acute treatments to the characteristics of their individual headaches.

In conclusion, our findings provide compelling real-world evidence that triptans are associated with better response rates than NSAIDs or APAP for the acute treatment of headaches in pwIIH. While this observation was particularly prominent in migraine-type attacks, it extended to other headache phenotypes in IIH as well, challenging aspects of the current phenotype-based treatment paradigm. Future prospective studies are needed to confirm these findings and establish evidence-based guidelines for acute headache treatment in IIH.

## Supplementary Information

Below is the link to the electronic supplementary material.

Supplementary Material 1

## Data Availability

Data supporting the findings of this study are available from the corresponding author upon reasonable request by a qualified researcher and upon approval by the ethics committee and the data-clearing committee of the Medical University of Vienna.
