# Supplementary material for: Acute headache treatment in idiopathic intracranial hypertension: treating to the phenotype?
Source: J Headache Pain. 2025 Nov 26;26(1):292. doi: 10.1186/s10194-025-02236-4 (PMC12750661; doi:10.1186/s10194-025-02236-4)
Supplement: Supplementary file 1 — Supplementary Material 1 [file 10194_2025_2236_MOESM1_ESM.docx]

**Appendix 1 - Statistical Analysis**

In the VIIH diary, people with idiopathic intracranial hypertension (pwIIH) typically report multiple headache attacks, which are treated as correlated repeated measures in the statistical analysis. Each individual is assumed to exert a random effect on treatment outcome, while each treatment exerts a conditional fixed effect based on the subject-specific random effect ^1^.

We employed a two-level nested logistic regression model to account for intra-subject covariance and concurrent medication use during the same headache attack.

Let Y*_ij_* denote the binary outcome for the *j*-th headache attack of the *i*-th pwIIH, where:

Y*_ij_* = 1 if the user reported “Helpful” and Y*_ij_* = 0 otherwise.

For a basic model evaluating the effect of a medication class or a single medication X, the logistic model is specified as:

𝑙𝑜𝑔𝑖𝑡(𝑃𝑟𝑜𝑏𝑎𝑏𝑖𝑙𝑖𝑡𝑦 𝑜𝑓 𝑌_𝑖𝑗_ = 1 ) = 𝛽_0_ + 𝛽_𝑖_+ 𝛾𝑋_𝑖𝑗_ + 𝜖_𝑖𝑗_

where:

- X_ij_ = 1 if class/medication X was used during the *j*-th attack by the *i*-th individual, and 0 otherwise,
- 𝛾 is the fixed effect representing the marginal efficacy of class/medication X,
- β_i_ is the subject-level random intercept capturing between-subject variability,
- ε_ij_ is the residual error term.

The logit function is defined as:

𝑙𝑜𝑔𝑖𝑡(𝑃𝑟𝑜𝑏𝑎𝑏𝑖𝑙𝑖𝑡𝑦 𝑜𝑓 𝑌_𝑖𝑗_ = 1) =𝑙𝑜𝑔 (𝑃𝑟𝑜𝑏𝑎𝑏𝑖𝑙𝑖𝑡𝑦 (𝑌_𝑖𝑗_ = 1) / 1 − 𝑃𝑟𝑜𝑏𝑎𝑏𝑖𝑙𝑖𝑡𝑦(𝑌𝑖𝑗 = 1))

To compare three classes of acute medications (i.e. triptans, APAP and NSAIDs) using NSAIDs as the reference group, or to compare eight specific substances using ibuprofen as the reference, the model expands to include indicator variables for each medication class or substance:

𝑙𝑜𝑔𝑖𝑡(𝑃𝑟𝑜𝑏𝑎𝑏𝑖𝑙𝑖𝑡𝑦 𝑜𝑓 𝑌_𝑖𝑗_ = 1) = 𝛽_0_ + 𝛽_𝑖_ + Σ𝛾_𝑘_ 𝑋_𝑘𝑖𝑗_ + 𝜖_𝑖𝑗_

Where: X_kij_ is an indicator variable for the k-th treatment option, 𝛾_k_ is the fixed effect comparing treatment k to the reference group (NSAID or ibuprofen), K=3 or K=8, depending on the comparison.

The estimated odds ratio (OR) for each treatment group X_k_ is calculated as:

𝑂𝑅(𝑋𝑘) = 𝑒𝑥𝑝 (𝛾_k_^)

To address potential confounding in treatment effectiveness, we applied propensity score weighting to adjust for the likelihood of receiving a specific medication. Propensity scores were estimated using a logistic regression model predicting the probability of application of each treatment based on baseline covariates including age, sex, and headache severity.

Inverse probability of treatment weights (IPTW) were computed and incorporated into the nested logistic regression models to balance the distribution of these covariates across treatment groups. This approach allows for a more unbiased estimate of treatment effects, approximating a pseudo-randomized comparison between treatments.

***Reference***

1. Marmura MJ, Silberstein SD, Schwedt TJ. The Acute Treatment of Migraine in Adults: The American Headache Society Evidence Assessment of Migraine Pharmacotherapies. Headache: J Head Face Pain. 2015;55:3–20.
